# Prognostic value of blood-based fibrosis biomarkers in patients with metastatic colorectal cancer receiving chemotherapy and bevacizumab

**DOI:** 10.1038/s41598-020-79608-0

**Published:** 2021-01-13

**Authors:** Neel I. Nissen, Stephanie Kehlet, Mogens K. Boisen, Maria Liljefors, Christina Jensen, Astrid Z. Johansen, Julia S. Johansen, Janine T. Erler, Morten Karsdal, Joachim H. Mortensen, Anette Høye, Nicholas Willumsen

**Affiliations:** 1 Biotech Research and Innovation Centre (BRIC), University of Copenhagen (UCPH), Copenhagen, Denmark; 2 Biomarkers and Research, Nordic Bioscience, Herlev Hovedgade 205-207, 2730 Herlev, Denmark; 3 Department of Oncology, Herlev and Gentofte Hospital, Copenhagen University Hospital, Copenhagen, Denmark; 4 Department of Clinical Science, Intervention and Technology, Karolinska University Hospital Huddinge, Stockholm, Sweden; 5 Department of Medicine, Herlev and Gentofte Hospital, Copenhagen University Hospital, Copenhagen, Denmark; 6 Department of Clinical Medicine, Faculty of Health and Medical Sciences, University of Copenhagen, Copenhagen, Denmark

**Keywords:** Cancer microenvironment, Tumour biomarkers, Colorectal cancer, Cancer

## Abstract

A desmoplastic colorectal cancer stroma, characterized by excess turnover of the cancer-associated fibroblast derived collagens type III and VI, can lead to reduced drug-uptake and poor treatment response. We investigated the association between biomarkers of collagen type III and VI and overall survival (OS) in patients with metastatic colorectal cancer (mCRC). Serum samples were collected from 252 patients with mCRC prior to treatment with bevacizumab and chemotherapy. Serum concentrations of biomarkers reflecting formation of collagen type III (PRO-C3) and VI (PRO-C6) and degradation of collagen type VI (C6M and C6Mα3) were determined by ELISA. The biomarkers were evaluated for associations with OS, individually, combined, and after adjusting for carcinoembryonic antigen (CEA), lactate dehydrogenase (LDH) and performance status (PS). High baseline levels (> median) of each collagen biomarker were significantly associated with shorter OS (PRO-C3: HR = 2.0, 95%CI = 1.54–2.63; PRO-C6: HR = 1.6, 95%CI = 1.24–2.11; C6M: HR = 1.4, 95%CI = 1.05–1.78; C6Mα3: HR = 1.6, 95%CI = 1.16–2.07). PRO-C3 and PRO-C6 remained significant after adjustment for CEA, LDH and PS. Weak correlations were seen between the collagen biomarkers (r = 0.03–0.59) and combining all improved prognostic capacity (HR = 3.6, 95%CI = 2.30–5.76). Collagen biomarkers were predictive of shorter OS in patients with mCRC. This supports that collagen- and CAF biology is important in CRC.

## Introduction

Screening programs and novel treatment strategies for colorectal cancer (CRC) have greatly improved survival during the last decade^1^. However, CRC is still the third most common cancer and the second most common cause of cancer-related death worldwide^2^. Patients diagnosed with early-stage disease can be treated with curative intent. But 20–25% of patients with CRC present with metastasis at diagnosis and the 5-year overall survival (OS) decrease drastically from 90% in stage I to 14% in stage IV^3^.

CRC can arise from multiple genetic and epigenetic pathways, which result in high tumour heterogeneity between patients^4,5^. Furthermore, the tumour microenvironment including non-mutant cells, fibroblasts, and the extracellular matrix (ECM) are major contributors to CRC progression^6,7^. Altogether, this affects treatment response and overall patient outcome, and the prognosis of patients with metastatic (m)CRC remains poor. The development of novel prognostic and predictive biomarkers, which can be used to monitor disease status and guide treatment decisions, is crucial.

During the last decade, biomarkers for CRC screening have been in focus with faecal occult blood testing as the most commonly used method. This tool is included in screening programs in western countries and has reduced the risk of CRC-associated mortality^4,8^. However, biomarkers for prediction of tumour aggressiveness, disease status, and treatment response are still lacking. Protein-based biomarkers, measured in liquid biopsies, have the potential to reflect a certain pathogenic phenotype. The local tumour microenvironment, including the ECM, could possess such biomarker targets, as it has been shown to play an important role in tumour initiation and progression^9^.

For many years, the ECM was believed to solely provide structural support for surrounding cells. However, it is now well recognized that it has an important function in regulating cell behaviour^6,7,9^. The ECM found in the intestines consists of a variety of proteins, including proteoglycans, elastin, laminins, and collagens. Overall, the ECM can be divided into two structures: the basement membrane (BM) underlining the epithelial and endothelial cells located in the mucosa and submucosa, and the interstitial matrix (IM), located underneath the BM in the lamina propria and submucosa^10^. The BM is primarily built up by collagen type IV, whereas the IM mainly consists of the fibroblast-derived collagens type I, III and VI^10^. Under healthy physiological states, the ECM is remodelled in an orderly and structured way. This homeostatic balance is disrupted in cancer pathologies. Here, the ECM becomes desmoplastic—a cancer-associated fibroblast (CAF) mediated uncontrolled ECM remodelling process, characterized by increased matrix-metalloproteinase (MMPs) activity, increased deposition of IM collagens and crosslinking of these collagens^6,9^. In CRC, the collagen fibres are thicker, denser, and more aligned compared to fibres in healthy tissue^11^. These events result in stiff tissue, which stimulates tumour growth, inflammation, angiogenesis, and enhances the metastatic potential^9,12–15^. Clinically, desmoplasia is associated with poor treatment response and short OS^9,12,16–20^. Two major desmoplasia-associated collagens are collagen type III and VI which are so-called fibrillar and beaded filaments, respectively. Collagen type III is expressed mainly in the lamina propria in the colon, whereas collagen type VI is localized in the crypt-villus axis in the interface between the BM and IM ^10^. Several studies have shown that collagen type III and VI promote tumour cell proliferation, angiogenesis, migration, metastasis, inflammation, and drug resistance^21–33^.

During tumour progression, the associated desmoplastic reaction generates collagen turnover fragments which are released to the circulation where they can act as a surrogate measure of desmoplasia and fibroblast activation. We and others have shown that several collagen biomarkers have a diagnostic, prognostic and predictive capacity in various solid tumour types^9,20,34–43^. As an example, turnover products from collagen type III are augmented in serum from patients with ovarian, melanoma, pancreatic and breast cancer^20–23,36,39,41,42^. In addition, biomarkers from collagen type III have been shown to predict response to treatment in patients with metastatic pancreatic cancer ^36^. Likewise, turnover products from collagen type VI have been shown to be increased in serum from patients with breast, colon, melanoma, ovary, prostate, lung and pancreatic cancer ^25,44–46^. We have shown that turnover products from collagen type I, III, and IV were increased in serum from patients with CRC when compared to levels in patients with adenomas and in healthy subjects^44,47^. The levels of these collagens were especially increased in later stages of CRC^47^. In this study, we evaluate serum biomarkers measuring turnover products of collagen type III and VI for their association with survival in patients with mCRC treated with standard of care chemotherapy and bevacizumab.

## Methods

### Patient samples

This study included pre-treatment serum samples from 252 patients with mCRC included in the biomarker study CREBB “ColoRectal cancer – Evaluation of Biomarkers in Bevacizumab treatment” from 2011 to 2016 at three Danish and four Swedish hospitals. The study included patients who were starting palliative treatment with standard chemotherapy and bevacizumab in any treatment line. All serum samples were drawn at baseline prior to bevacizumab + chemotherapy treatment. The study was carried out in accordance with the recommendations of the Danish Regional Committee on Health Research Ethics. The CREBB protocol was approved by the Danish Regional Committee on Health Research Ethics (Approval ID: H-3-2010-121) and the Data Protection Agency (Approval ID: 2007-58-0015/HEH.750.24-44). All subjects gave written informed consent in accordance with the Declaration of Helsinki. Nordic Bioscience received anonymized serum samples and clinical data. Clinical data included: Age, gender, number of metastatic sites at study inclusion, performance status (PS), line of treatment, primary tumour resected or in situ at baseline, baseline carcinoembryonic antigen (CEA, cut off: 5 ug/L), baseline lactate dehydrogenase (LDH, cut off: 205 U/L), baseline platelets (cut off: 390 × 10^9^/L), baseline neutrophils (cut off: 5.9 × 10^9^/L) and baseline white blood cells (cut off: 8.8 × 10^9^/L). Demographics and clinical profiles are shown in Table 1.

**Table 1:** Patient demographics and clinical profile.

Clinical variables	Study population (n = 252)
**Age, (years)**	
Median (min, max)	67 (28–99)
**Gender, n (%)**	
Male	154 (61%)
Female	98 (39%)
**Number of metastatic sites, n (%)**	
1 site	96 (38%)
> 1 site	156 (62%)
**Liver metastasis**	
Yes	157 (62%)
No	94 (37%)
Unknown	1 (< 1%)
**Performance status, n (%)**	
0	165 (65%)
1	72 (29%)
≥ 2	19 (4%)
Unknown	6 (2%)
**Line of palliative chemotherapy, n (%)**	
First	132 (52%)
Second or later	120 (48%)
Primary tumor resected at baseline, n (%)	138 (55%)
**Carcinoembryonic antigen, n (%)**	
≤ 5 ug/L	35 (14%)
> 5 ug/L	150 (59%)
Unknown	67 (27%)
**Lactate dehydrogenase, n (%)**	
≤ 205 U/L	104 (41%)
> 205 U/L	124 (49%)
Unknown	24 (10%)
**Platelets, n (%)**	
≤ 390 × 10^9^/L	216 (86%)
> 390 × 10^9^/L	31 (12%)
Unknown	5 (2%)
**Neutrophils, n (%)**	
≤ 5.9 × 10^9^/mL	146 (58%)
> 5.9 × 10^9^/mL	51 (20%)
Unknown	55 (22%)
**White blood cells, n (%)**	
≤ 8.8 × 10^9^/mL	190 (75%)
> 8.8 × 10^9^/mL	57 (23%)
Unknown	5 (2%)

### Biomarker measurements

**Figure 1:**
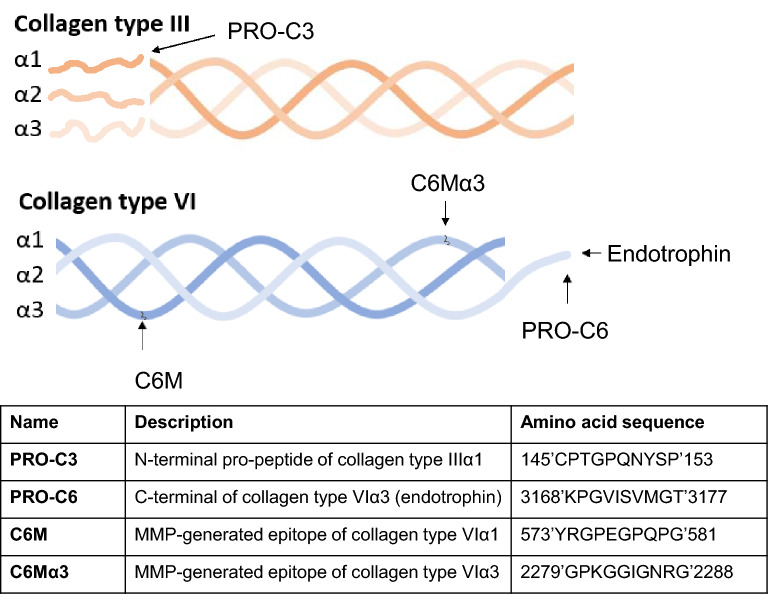
Schematic illustration of collagen type III and type VI with descriptions of their respective biomarker targets.

Four different collagen turnover fragments (biomarkers) were measured in serum samples, drawn prior to bevacizumab + chemotherapy treatment, using competitive enzyme-linked immunosorbent assays (ELISA). The assays measured the N-terminal pro-peptide of collagen type IIIα1 (PRO-C3), the C-terminal (endotrophin) of collagen type VIα3 (PRO-C6), a MMP-generated epitope of collagen type VIα1 (C6M) and a MMP-generated epitope of collagen type VIα3 (C6Mα3). Description and visualization of the biomarker targets are shown in Fig. 1. The assays were run according to manufacturer’s instructions (Nordic Bioscience, Herlev, Denmark). Briefly, 96-well streptavidin-plates were coated with 100 μL biotinylated synthetic target-peptide dissolved in assay buffer. The plates were incubated for 30 min at 20 °C. After incubation, the plates were washed five times in wash buffer (20 mM Tris, 50 mM NaCl, pH 7.2). Next, 20 μL of standard peptide and pre-diluted samples were added to the appropriate wells followed by 100 μL of peroxidase-conjugated specific monoclonal antibody and incubated at 1 h at 20 °C or 20 h at 4 °C. After incubation, plates were washed five times with wash-buffer and 100 μL of tetramethylbenzinidine (cat. 438OH, Kem-En-Tec diagnostics, Denmark) was added to each well. Plates were incubated for 15 min at 20 °C followed by the addition of 100 μL 0.18 M H_2_SO_4_ to stop the enzymatic reaction. All incubations were performed on a plate shaker at 300 rpm in darkness. The absorbance (optical density, OD) was measured at 450 nm with 650 nm as reference. A calibration curve was plotted using a 4-parameter logistic curve fit.

### Statistical analysis

Results are reported in accordance with the REMARK (Reporting Recommendations for Tumor Marker Prognostic Studies) guidelines ^48^. Spearman correlation coefficient was used to calculate correlations between PRO-C3, PRO-C6, C6M and C6Mα3. Kaplan–Meier curves were used to assess the relationship between high (> median) and low (≤ median) collagen biomarker levels and OS, individually and in biomarker combinations. A univariate Cox proportional-hazards regression model was used to calculate the hazard ratios (HR) with 95% Cl for the OS per biomarker and clinical co-variates: Age, gender, number of metastatic sites, PS, line of treatment, primary tumour in situ or resected, serum CEA (cut-off: 5 ug/L), serum LDH (cut-off: 205 U/L), platelets (390 × 10^9^/L), neutrophils (5.9 × 10^9^/L), and white blood cells (8.8 × 10^9^/L). A multivariate Cox proportional-hazard regression model including PS, primary tumour in situ or resected, CEA, LDH and cell counts was used to evaluate potential independent predictive value of the collagen biomarkers. A *p*-value of *p* < 0.05 was considered statistically significant. Graph design and statistical analyses were performed using GraphPad Prism Version 8.2 (GraphPad Software, Inc.) and MedCalc version 14 (Medcalc Software).

### Ethical approval and consent to participate

The CREBB protocol was approved by the Danish Regional Committee on Health Research Ethics (Approval ID: H-3-2010-121) and the Data Protection Agency (Approval ID: 2007–58-0015 / HEH.750.24-44). All subjects gave written informed consent in accordance with the Declaration of Helsinki.

## Results

### Biomarkers reflecting the turnover of collagen type III and VI are elevated in patients with mCRC

**Figure 2:**
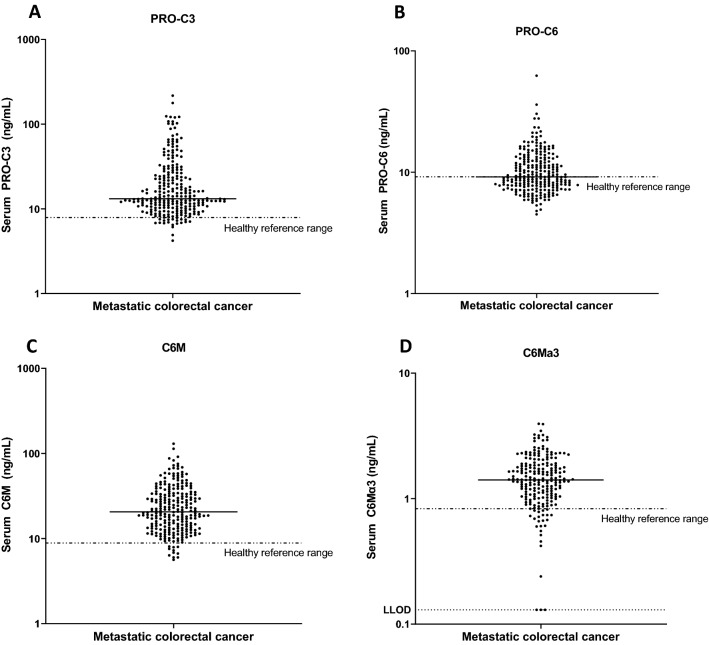
Serum levels of four different biomarkers reflecting (**A**) formation of collagen type III (PRO-C3), (**B**) formation of collagen type VI (PRO-C6), (**C**) degradation of collagen type VIα1 (C6M) and (**D**) degradation of collagen type VIα3 (C6Mα3) in patients with metastatic colorectal cancer with their respective reference range from healthy controls (dotted line). Black line, median. LLOD; lower limit of detection.

Figure 2 shows the distribution of the serum biomarkers PRO-C3 (n = 252), PRO-C6 (n = 252), C6M (n = 252) and C6Mα3 (n = 212) from patients with mCRC. Most biomarkers were elevated compared to previously reported healthy reference ranges^41,44^ with median biomarker levels approximately two-fold higher than the reference range. Patient to patient variation was observed in absolute biomarker levels ranging from PRO-C3: 4.2–216 ng/mL (median 13.2 ng/mL), PRO-C6: 4.5–62.5 ng/mL (median 9.2 ng/mL), C6M: 5.6–130 ng/mL (median 20.6 ng/mL) and C6Mα3: 0.13–3.9 ng/mL (median 1.4 ng/mL).

### Biomarkers reflecting the turnover of collagen type III and VI are associated with overall survival in patients with mCRC

**Figure 3:**
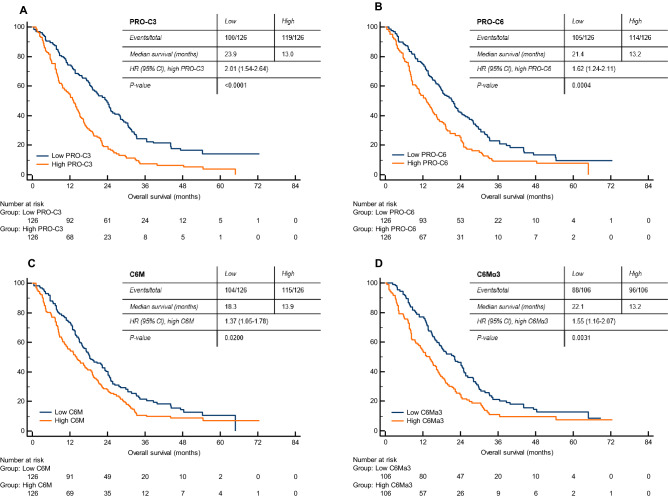
Assessment of the prognostic potential of low and high (median) (**A**) PRO-C3 (formation of collagen type III), (**B**) PRO-C6 (formation of collagen type VI), (**C**) C6M (degradation of collagen type VIα1) and (**D**) C6Mα3 (degradation of collagen type VIα3) serum levels by Kaplan Meier plots. Cox proportional-hazards regression was used to calculate the hazard ratios (HR) with 95% Cl and *p*-values. A *p* < 0.05 was considered significant.

To investigate the association between the turnover of collagen type III and VI and OS in patients with mCRC, we assessed the prognostic potential of PRO-C3, PRO-C6, C6M and C6Mα3 by Kaplan–Meier curves and univariate Cox proportional-hazard models. The median OS for the cohort was 19.8 months (range, 0.1–72.5 months). Firstly, we assessed the prognostic value of the biomarkers PRO-C3 and PRO-C6 reflecting formation of collagen type III and VI. Kaplan–Meier curves for PRO-C3 showed that high levels (above median) were significantly associated with poor OS (*p* < 0.0001, HR 2.0, 95% CI 1.54–2.64) (Fig. 3A). Median OS for low levels of PRO-C3 was 23.9 months and high levels of PRO-C3 was 13.0 months (Fig. 3A). High levels (above median) of PRO-C6 were also significantly associated with short OS (*p* = 0.0004, HR 1.6, 95% CI 1.24–2.11) (Fig. 3B). Median OS for low levels of PRO-C6 was 21.4 months versus 13.2 months for high levels of PRO-C6 (Fig. 3B). Next, we evaluated the prognostic value of the biomarkers C6M and C6Mα3 reflecting degradation of collagen type VI. High levels of C6M (above median) were significantly associated with poor OS (*p* = 0.02, HR 1.4, 95% CI 1.05–1.78) (Fig. 3C). Patients with low levels of C6M had a median OS at 18.3 months compared to 13.9 months for patients with high levels of C6M (Fig. 3C). Short OS was also significantly associated with high levels of C6Mα3 (*p* = 0.003, HR 1.6, 95% CI 1.16–2.07) (Fig. 3D). Median OS for low levels of C6Mα3 was 22.1 months and high levels of C6Mα3 was 13.2 months (Fig. 3D). To evaluate if the association of the collagen biomarkers with OS were independent of clinical co-variates, a multivariate Cox analysis was performed adjusting each collagen biomarkers for PS, primary tumour resected or in situ, CEA, LDH, platelets and neutrophils. All biomarkers were still predictive of short OS when adjusting for PS and primary tumour resected or in situ*.* High levels of C6M and C6Mα3 were not independent of CEA, LDH and cell counts. However, PRO-C3 and PRO-C6 were still predictive of short OS when adjusting for these clinical co-variates (Table 2). Since approximately 50% of cases underwent other chemotherapy regimens prior to the bevacizumab-containing regimen, patients were divided into first and second or later line of chemotherapy treatment. Here the relationship between biomarker levels high (> median) and low (≤ median) and OS were assessed. The same trends for the biomarkers, i.e. high biomarker levels associated with poor OS, were seen in both first and second or later line of chemotherapy treatment (Supplementary Fig S1 and S2).

**Table 2:** Association between biomarker levels, clinical covariates and outcome for patients with mCRC. Uni- (a) and multivariate (b) cox proportional-hazards regression were used to calculate the hazard ratios (HR) with 95% Cl and *p*-values.

Variable		Overall survival		
**a**				
**Univariate analysis**				
	Univariate analysis	HR	95% Cl	P-value
PRO-C3	Continuous	1.01	1.00–1.01	< *0.0001*
	High (> median) versus low (≤ median)	2.01	1.54–2.64	< *0.0001*
PRO-C6	Continuous	1.02	1.00–1.04	*0.0207*
	High (> median) versus low (≤ median)	1.62	1.24–2.11	*0.0004*
C6M	Continuous	1.01	1.00–1.02	*0.0076*
	High (> median) versus low (≤ median)	1.37	1.05–1.78	*0.0200*
C6Mα3	Continuous	1.30	1.07–1.61	*0.0180*
	High (> median) versus low (≤ median)	1.55	1.16–2.07	*0.0031*
Age, per year increase	Continuous	1.01	1.00–1.03	0.0708
Gender	Female versus male	1.14	0.87–1.49	0.8421
Number of metastatic sites	> 1 versus 1	1.24	0.94–1.63	0.1186
Liver metastasis	Yes versus no	1.07	0.81–1.14	0.6191
Performance status	1 versus 0	1.69	1.25–2.28	*0.0006*
	* ≥ 2* versus 0	3.04	1.54–6.00	*0.0014*
Line of treatment	Second or later versus first	1.11	0.85–1.45	0.4246
Primary resected versus in situ at baseline		0.72	0.55–0.94	*0.0155*
CEA	High (> 5 ug/L) versus low (≤ 5 ug/mL)	1.70	1.12–2.53	*0.0089*
LDH	High (> 205 U/L) versus low (≤ 205 U/mL)	2.13	1.59–2.84	< *0.0001*
Platelets	High (> 390 × 10^9^ cells/L) versus low (390 × 10^9^ cells/L))	1.70	1.15–2.51	*0.0086*
Neutrophils	High (> 5.9 × 10^9^ cells/L) versus low (5.9 × 10^9^ cells/L)	1.66	1.18–2.33	*0.0037*
White blood cells	High (> 8.8 × 10^9^ cells/L) vs. low (≤ 8.8 × 10^9^ cells/L)	1.74	0.43–7.01	0.4378

Variables	HR	95% Cl	*P* value	
**b**				
**Multivariate analysis**				
**Adjusted for PS and primary tumor resected or in situ**				
PRO-C3 (high vs. low)	1.88	1.43–2.48	< *0.0001*	
PRO-C6 (high vs. low)	1.51	1.14–2.00	*0.0041*	
C6M (high vs. low)	1.33	1.01–1.76	*0.0411*	
C6Mα3 (high vs. low)	1.44	1.07–1.94	*0.0158*	
**Adjusted for CEA and LDH**				
PRO-C3 (high vs. low)	1.60	1.15–2.22	*0.0058*	
PRO-C6 (high vs. low)	1.48	1.08–2.04	*0.0162*	
C6M (high vs. low)	1.34	0.97–1.86	0.0722	
C6Mα3 (high vs. low)	1.30	0.90–1.90	0.1664	
**Adjusted for platelets and neutrophils**				
PRO-C3 (high vs. low)	1.96	1.44–2.69	< *0.0001*	
PRO-C6 (high vs. low)	1.74	1.28–2.36	*0.0004*	
C6M (high vs. low)	1.30	0.95–1.75	0.1057	
C6Mα3 (high vs. low)	1.56	1.11–2.20	*0.0103*	
**Adjusted for PS, primary tumor resected or in situ, CEA, LDH, platelets and neutrophils**				
PRO-C3 (high vs. low)	1.43	0.98–2.08	0.0623	
PRO-C6 (high vs. low)	1.71	1.18–2.48	*0.0043*	
C6M (high vs. low)	1.23	0.86–1.76	0.2481	
C6Mα3 (high vs. low)	1.11	0.71–1.72	0.6481	

A p-value of *P* < 0.05 was considered statistically significant (shown in italics).

PS; performance status. CEA; baseline carcinoembryonic antigen. LDH; baseline lactate dehydrogenase.

Biomarker high/low are based on median.

### Combination of collagen biomarkers has an additive effect on the prognostic value

**Figure 4:**
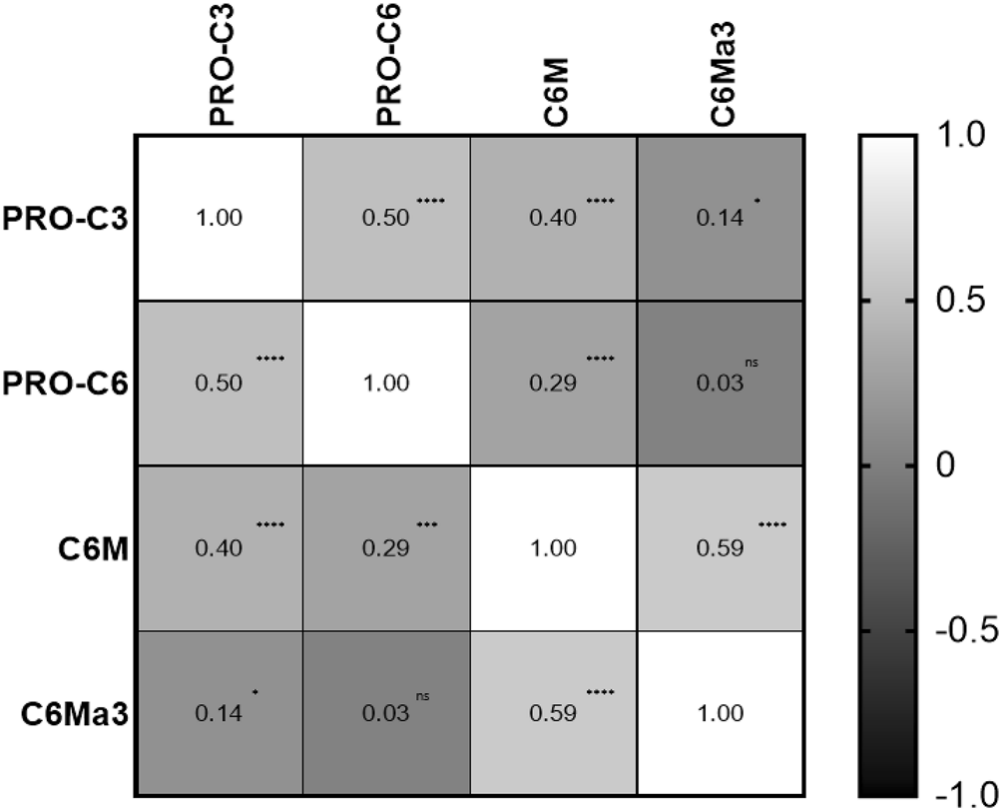
Correlation between biomarker levels where compared pairwise and evaluated by the nonparametric Spearman correlation coefficient. ns, not significant. *p < 0.05. **p < 0.01. ***p < 0.001. ****p < 0.0001. A *p* < 0.05 was considered significant.

Since all four collagen biomarkers were individually prognostic for OS, we wanted to investigate the relationship between the markers. We therefore correlated the markers using a nonparametric Spearman correlation coefficient. There was a significant but modest correlation between PRO-C6 versus PRO-C3, C6Mα3 versus C6M and C6M versus PRO-C3. Furthermore, there was a significant but weak correlation between C6Mα3 versus PRO-C3 and C6M versus PRO-C6. There was no significant correlation between C6Mα3 and PRO-C6 (Fig. 4).

**Figure 5:**
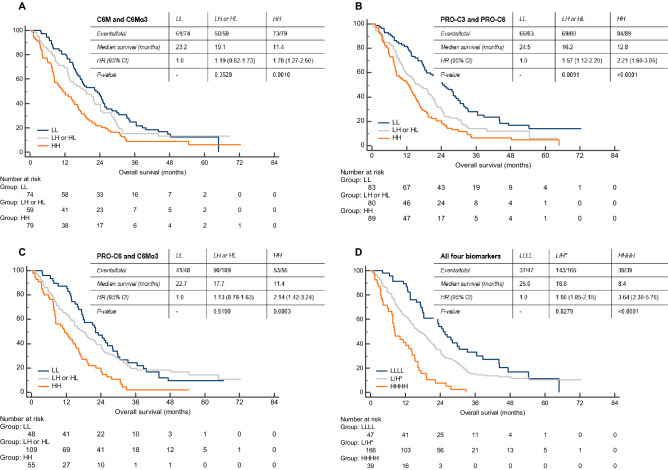
Assessment of the prognostic potential of combining the different biomarkers by Kaplan Meier plots. (**A**) LL; low C6M and low C6Mα3, LH or HL; low C6M and high C6Mα3 or high C6M and low C6Mα3, HH; high C6M and high C6Mα3. (**B**) LL; low PRO-C3 and low PRO-C6, LH or HL; low PRO-C3 and high PRO-C6 or high PRO-C3 and low PRO-C6, HH; high PRO-C6 and high PRO-C6. (**C**) LL; low PRO-C6 and low C6Mα3, LH or HL; low PRO-C6 and high C6Mα3 or high PRO-C6 and low C6Mα3, HH; high PRO-C6 and high C6Mα3. (**D**) LLLL; low PRO-3, PRO-C6, C6M and C6Mα3, L/H*; at least one low or high PRO-C3, PRO-C6, C6M or C6Mα3, HHHH; high PRO-C3, PRO-C6, C6M and C6Mα3. Univariate Cox proportional-hazards regression was used to calculate the hazard ratios (HR) with 95% Cl and *p*-values. A *p* < 0.05 was considered significant.

As there was only a modest correlation between the markers, we evaluated the prognostic capacity of combining the markers. Firstly, we combined the two degradation markers C6M and C6Mα3. Patients were divided into three groups: low C6M and low C6Mα3, low or high C6M/C6Mα3 and high C6M and high C6Mα3. OS for patients with all low biomarker levels was 23.2 months compared to 11.4 months for patients with all high biomarker levels (Fig. 5A). High biomarker levels were significantly associated with short OS (low/high or high/low versus low/low: *p* = 0.3528, HR 1.19, 95% CI 0.82–1.73. High/high versus low/low: *p* = 0.0010, HR 1.78, 95% CI 1.27–2.50) (Fig. 5A). Comparable results were seen when combining the two formation markers PRO-C3 and PRO-C6 as described above. Again, high levels were significantly associated with short OS (low/high or high/low versus low/low: *p* = 0.0091 h 1.57, 95% CI 1.12–2.20. High/high versus low/low: *p* < 0.0001, HR 2.21, 95% CI 1.60–3.06) (Fig. 5B). Median OS for patients with low biomarker levels was 24.5 months compared to 12.8 months for patients with high biomarker levels (Fig. 5B). Next, we combined PRO-C6 and C6Mα3, two different biomarkers targeting the alpha3 chain on type VI collagen. Patients with high levels of both biomarkers had a significant shorter OS than patients with low levels (low/high or high/low versus low/low: *p* = 0.5190, HR 1.13, 95% CI 0.87–1.63. High/high versus low/low: *p* = 0.0003, HR 2.14, 95% CI 1.42–3.24) (Fig. 5C). Median OS for patients with low biomarker levels was 22.7 months compared to 11.4 months for patients with high biomarker levels (Fig. 5C). Finally, when combining all the markers the patients with high biomarker levels had a significant shorter OS compared to patients with low biomarker levels (low/high or high/low versus low/low: *p* = 0.0279, HR 1.50, 95% CI 1.05–2.15. High/high versus low/low: *p* < 0.0001, HR 3.64, 95% CI 2.30–5.76) (Fig. 5D). Median OS for patients with low biomarker levels was 25.0 months compared to 8.4 months for patients with high biomarker levels (Fig. 5D). These results show that combining the different biomarkers, i.e. assessing the turnover of different collagens, improves the prognostic value.

## Discussion and conclusion

Biomarkers reflecting the turnover of collagen type III and VI are elevated in patients with CRC, suggesting that this is a pathological feature in CRC^44,47^. However, much less is known about the prognostic value of using fibrosis biomarkers in CRC and in the metastatic setting where mainly palliative care is an option.

In the present study, we assessed the ability of four serum biomarkers, reflecting the turnover of collagen type III and VI for predicting OS in patients with mCRC before palliative treatment with bevacizumab combined with standard chemotherapy. The results indicate that high levels of biomarkers reflecting collagen III (PRO-C3) and VI (PRO-C6) formation and collagen type VI degradation (C6M and C6Mα3) is predictive of poor survival in patients with mCRC. Furthermore, combining these formation and degradation biomarkers, i.e. measuring the turnover of two different collagens, have an additive effect on the prognostic value.

Desmoplasia is an important cancer hallmark in most cancer pathologies^9^. In support, several clinical studies have shown that desmoplasia is associated with poor treatment response and shorter survival in various solid tumour types^9,12,16–20^. In CRC however, desmoplasia is a rather controversial topic. Overall, studies report contradicting results showing that desmoplasia is a complex and heterogeneous reaction^6,20,49–52^. Nonetheless, we and others have shown that collagen fragments, including PRO-C3 and PRO-C6, are increased in serum from CRC patients, and that the levels of these fragments increase in later stages of disease^43,44,47,53–55^. In this study, we show that collagen fragments are expressed in same levels as reported before in CRC^38,44^. Taken together, these data suggest that desmoplasia and turnover of collagen are pathological features of mCRC associated with a poor prognosis. Interestingly, the formation of collagen type III and VI are not increased in inflammatory bowel diseases such as Crohn’s disease ^56^ further suggesting that an increased turnover of these collagens might be cancer specific.

Supporting the present findings, biomarkers reflecting degradation and formation of collagen can possibly be a useful prognostic tool for patients with solid tumours. We have previously demonstrated that biomarkers reflecting collagen turnover are predictive of survival in malignant melanoma, breast and pancreatic cancer^20,36,41,42,57^. Currently, serum CEA is the most common molecular biomarker used in the clinic to asses disease status in CRC^58–60^. CEA is often elevated in serum from patients with various cancers and associates with severity of disease, though with low sensitivity and specificity^58^. Interestingly, the prognostic values of PRO-C3 and PRO-C6 reported here were independent of CEA. CEA is a measure related to cancer cells, whereas PRO-C3 and PRO-C6 reflect different biology that are more directly linked to desmoplasia and fibroblast activity^9^.

In cancer pathologies, collagen type III and VI are produced by CAFs and are not just structural proteins but a major part of the desmoplastic reaction^9^. In vitro studies of collagen type III have demonstrated that cancer cells stimulated with this collagen have decreased E-cadherin expression, increased proliferation rate, and augmented migratory capacity^21–23^. Collagen type VI is also known for its pro-tumorigenic signalling capacity. The C5 domain of the collagen type VIα3 chain, also called endotrophin, has been found to promote metastasis, tumour cell proliferation, inflammation, and cisplatin resistance in cancer cell lines and tumour mouse models^24–33^. In addition, endotrophin is believed to contribute to angiogenesis^25,27,28^. All patients in the present study were treated with bevacizumab, a monoclonal antibody targeting vascular-endothelial growth factor (VEGF). By its binding to VEGF, bevacizumab is thought to decrease new blood vessel formation and tumour growth^61^. Bevacizumab, in combination with chemotherapy, has shown to improve survival in patients with mCRC and is approved for use in a range of cancer indications^61^. It could be argued that collagen signalling fragments, such as endotrophin, could have counteractive effects on anti-VEGF targeting drugs. Thus, it could be possible that patients with high levels of PRO-C6, i.e. containing high levels of endotrophin, responds poorly to bevacizumab. If this is the case, PRO-C6 could be used in the clinic to find patients responding to bevacizumab prior to treatment.

In addition, it is thought that the desmoplastic reaction can result in a stromal barrier creating a structural hindrance for drug uptake in the cancer cells^62^. Mariathasan et. al. showed that T-cells can be trapped in the collagen assembly, thereby reducing the effect of immunotherapies^63^. Augmented levels of collagen biomarkers could reflect a stromal barrier and explain the poor prognosis of patients with mCRC. In an era of precision medicine, it is important to develop biomarkers that can predict response to therapy, which collagen biomarkers have the potential to do. Recently, biomarkers reflecting turnover of collagen type III have been shown to predict which patients responded to anti-stromal therapy in the pancreatic cancer setting^36^. Likewise, it could be speculated that anti-stromal therapy in CRC could improve prognosis further. Anti-stromal therapy, such as anti-TGFß compounds, has been under high attention during the last decade^64^. Collagen biomarkers have the potential to be used to asses mode of action and to predict patients responding to these classes of drugs. Furthermore, future studies are needed to investigate the biomarker prediction values for patients divided into specific treatment regimens.

Clearly, collagen is not just collagen, and a combination of collagen biomarkers could have the potential to provide additive prognostic and predictive value, as has also been shown for the combination of CEA and CA19-9 in CRC^59,60^. We demonstrated that by quantifying the turnover of different collagen fragments, not simply measuring either formation or degradation of collagens, it can provide an additive effect on the prognostic value. Patients with mCRC with low levels of all four collagen biomarkers survived three times longer compared to patients with high levels. This suggests, that desmoplasia is not just characterized by either an increased formation or an increased degradation of collagen, but merely an unbalanced and chaotic turnover of several collagens leading to poor patient outcome. Furthermore, the combination between biomarkers of the same alpha chains (i.e. PRO-C6 and C6Mα3) resemble an important lesson to be learned, namely that targeting specific neo-epitopes on a protein provide additional value rather than looking at the total pool of that one alpha chain either by conventional protein- and gene expression analyses. This observation could be explained by a differential biology reflected in the specific neo-epitopes where PRO-C6 may be reflective of increased CAF activity and C6Mα3 may be reflective of an increased MMP-activity and enhanced inflammatory response. To confirm the relationship between PRO-C6 and CAFs, it would be interesting, in future studies, to correlate PRO-C6 and the other biomarkers, measured in this study, with the different consensus molecular subtypes (CMS), as CMS4 has shown to be of high CAF content and respond poorly to bevacizumab^65–68^. Unfortunately, no histological data on CMS subtypes was available in this cohort of patients.

The present study needs to be validated prospectively in larger well-characterized cohorts. Of the four biomarkers, PRO-C3 performed best in predicting survival outcome in patients with mCRC independent of other risk factors. PRO-C3 is a well-known liver fibrosis marker, and since the most common metastatic site in CRC is the liver, this could possibly drive the high levels of PRO-C3 seen in this study^69^. In this study, OS was not dependent on liver metastasis. Furthermore, in a large cohort of patients with pancreatic cancer, where the liver is also the primary metastatic site, the prognostic value of PRO-C3 was also found to be independent of liver metastasis^41^.

In summary, we find that biomarkers measuring the turnover of collagen type III and VI, i.e. desmoplasia and CAF activity, are predictive of shorter OS in patients with mCRC. This suggests an association between tumour fibrosis and response to treatment in patients with mCRC. Furthermore, fibrosis biomarkers might have the potential to be used to monitor disease status, treatment response and in tailoring treatment strategies in CRC.

## Supplementary Information

Supplementary Information.

## Data Availability

The datasets used and/or analysed during the current study are available from the corresponding author on reasonable request.

## Competing of interests

S.K., C.J., M.K., J.M. and N.W. are employed at Nordic Bioscience involved in the discovery and development of serological biomarkers.

## References

[1] Miller EA, Pinsky PF, Schoen RE, Prorok PC, Church TR (2019). Effect of flexible sigmoidoscopy screening on colorectal cancer incidence and mortality: long-term follow-up of the randomised US PLCO cancer screening trial. Lancet Gastroenterol. Hepatol..

[2] World Health Organization. *WHO Report on Cancer: setting prorities, investing wisely and providing care for all*. https://www.who.int/publications-detail/who-report-on-cancer-setting-priorities-investing-wisely-and-providing-care-for-all (2020).

[3] Siegel RL (2017). Colorectal cancer statistics, 2017. CA. Cancer J. Clin..

[4] Dickinson BT, Kisiel J, Ahlquist DA, Grady WM (2015). Molecular markers for colorectal cancer screening. Gut.

[5] Dekker E, IJspeert JEG (2018). Serrated pathway: A paradigm shift in CRC prevention. Gut.

[6] Crotti S (2017). Extracellular matrix and colorectal cancer: how surrounding microenvironment affects cancer cell behavior?. J. Cell. Physiol..

[7] Bhome R (2016). Translational aspects in targeting the stromal tumour microenvironment: From bench to bedside. New Horizons Transl. Med..

[8] Alves Martins BA (2019). Biomarkers in Colorectal Cancer: The Role of Translational Proteomics Research. Front. Oncol..

[9] Nissen NI, Karsdal M, Willumsen N (2019). Collagens and Cancer associated fibroblasts in the reactive stroma and its relation to Cancer biology. J. Exp. Clin. Cancer Res..

[10] Mortensen J (2019). The intestinal tissue homeostasis: the role of extracellular matrix remodeling in inflammatory bowel disease. Expert Rev. Gastroenterol. Hepatol..

[11] Birk JW (2014). Second harmonic generation imaging distinguishes both high-grade dysplasia and cancer from normal colonic mucosa. Dig. Dis. Sci..

[12] Pickup MW, Mouw JK, Weaver VM (2014). The extracellular matrix modulates the hallmarks of cancer. EMBO Rep..

[13] Calvo F (2013). Mechanotransduction and YAP-dependent matrix remodelling is required for the generation and maintenance of cancer-associated fibroblasts. Nat. Cell Biol..

[14] Heldin CH, Rubin K, Pietras K, Östman A (2004). High interstitial fluid pressure—an obstacle in cancer therapy. Nat. Rev. Cancer.

[15] Fang M, Yuan J, Peng C, Li Y (2014). Collagen as a double-edged sword in tumor progression. Tumor Biol..

[16] Hanley CJ (2016). A subset of myofibroblastic cancer-associated fibroblasts regulate collagen fiber elongation, which is prognostic in multiple cancers. Oncotarget.

[17] Kashima H (2019). Cancer-associated fibroblasts (CAFs) promote the lymph node metastasis of esophageal squamous cell carcinoma. Int. J. Cancer.

[18] McCarthy JB, El-Ashry D, Turley EA (2018). Hyaluronan, Cancer-Associated Fibroblasts and the Tumor Microenvironment in Malignant Progression. Front. Cell Dev. Biol..

[19] LeBleu VS, Kalluri R (2018). A peek into cancer-associated fibroblasts: origins, functions and translational impact. Dis. Model. Mech..

[20] Jensen C (2018). Non-invasive biomarkers derived from the extracellular matrix associate with response to immune checkpoint blockade (anti-CTLA-4) in metastatic melanoma patients. J. Immunother. Cancer..

[21] Chintala S, Sawaya R, Gokaslan Z, Rao J (1996). The effect of type III collagen on migration and invasion of human. Cancer Lett..

[22] Hirai K, Shimada H, Ogawa T, Taji S (1991). The spread of human lung cancer cells on collagens and its inhibition by type III collagen. Clin Exp Metastasis.

[23] Menke A (2001). Down-regulation of E-cadherin gene expression by collagen type I and Type III in pancreatic cancer cell lines 1. Biochemistry.

[24] Cescon M, Gattazzo F, Chen P, Bonaldo P (2015). Collagen VI at a glance. J. Cell Sci..

[25] Chen P, Cescon M, Bonaldo P (2013). Collagen VI in cancer and its biological mechanisms. Trends Mol. Med..

[26] Park J, Morley TS, Scherer PE (2013). Inhibition of endotrophin, a cleavage product of collagen VI, confers cisplatin sensitivity to tumours. EMBO Mol. Med..

[27] Park J, Scherer PE (2012). Endotrophin: linking obesity with aggressive tumor growth. Oncotarget.

[28] Schnoor M (2008). Production of type VI collagen by human macrophages: a new dimension in macrophage functional heterogeneity. J. Immunol..

[29] Wright A, Li Y-H, Zhu C, Woodruff W, Coulter H (2008). The differential effect of endothelial cell factors on *in vitro* motility of malignant and non-malignant cells. Ann Biomed Eng.

[30] Han J, Daniel JC (1995). Biosynthesis of type VI collagen by glioblastoma cells and possible function in cell invasion of three-dimensional matrices. Connect. Tissue Res..

[31] Sherman-Baust CA (2003). Remodeling of the extracellular matrix through overexpression of collagen VI contributes to cisplatin resistance in ovarian cancer cells. Cancer Cell.

[32] Varma RR (2005). Gene expression profiling of a clonal isolate of oxaliplatin-resistant ovarian carcinoma cell line A2780/C10. Oncol. Rep..

[33] Iyengar P (2005). Adipocyte-derived collagen VI affects early mammary tumor progression in vivo, demonstrating a critical interaction in the tumor/stroma microenvironment. J. Clin. Invest..

[34] Willumsen N (2013). Extracellular matrix specific protein fingerprints measured in serum can seperate pancreatic cancer patients from healthy controls. BMC Cancer.

[35] Willumsen N, Thomsen LB, Bager CL, Jensen C, Karsdal MA (2018). Quantification of altered tissue turnover in a liquid biopsy: a proposed precision medicine tool to assess chronic inflammation and desmoplasia associated with a pro-cancerous niche and response to immuno-therapeutic anti-tumor modalities. Cancer Immunol. Immunother..

[36] Wang S (2018). Extracellular matrix (ECM) circulating peptide biomarkers as potential predictors of survival in patients (pts) with untreated metastatic pancreatic ductal adenocarcinoma (mPDA) receiving pegvorhyaluronidase alfa (PEGPH20), nab-paclitaxel (A), and gemcita. J. Clin. Oncol..

[37] Bager CL (2016). Remodeling of the tumor microenvironment predicts increased risk of cancer in postmenopausal women: The prospective epidemiologic risk factor (PERF I) study. Cancer Epidemiol. Biomarkers Prev..

[38] Willumsen N (2017). Excessive matrix metalloprotease-mediated degradation of interstitial tissue (type I collagen) independently predicts short-term survival in an observational study of postmenopausal women diagnosed with cancer. Oncotarget.

[39] Bager CL (2015). Collagen degradation products measured in serum can separate ovarian and breast cancer patients from healthy controls: a preliminary study. Cancer Biomarkers.

[40] Willumsen N (2014). Serum biomarkers reflecting specific tumor tissue remodeling processes are valuable diagnostic tools for lung cancer. Cancer Med..

[41] Chen IM (2020). Clinical value of serum hyaluronan and propeptide of type III collagen in patients with pancreatic cancer. Int. J. Cancer.

[42] Willumsen N (2019). Collagen fragments quantified in serum as measures of desmoplasia associate with survival outcome in patients with advanced pancreatic cancer. Sci. Rep..

[43] Giussani M, Triulzi T, Sozzi G, Tagliabue E (2019). Tumor extracellular matrix remodeling: new perspectives as a circulating tool in the diagnosis and prognosis of solid tumors. Cells.

[44] Willumsen N, Bager C, Karsdal MA (2019). Matrix metalloprotease generated fragments of type VI collagen have serum biomarker potential in cancer – a proof of concept study. Transl. Oncol..

[45] Burchardt ER, Hein R, Bosserhoff AK (2003). Laminin, hyaluronan, tenascin-C and type VI collagen levels in sera from patients with malignant melanoma. Clin. Exp. Dermatol..

[46] Kang CY (2014). Clinical Significance of Serum COL6A3 in Pancreatic Ductal Adenocarcinoma. J. Gastrointest. Surg..

[47] Kehlet SN (2016). Excessive collagen turnover products are released during colorectal cancer progression and elevated in serum from metastatic colorectal cancer patients. Sci. Rep..

[48] McShane LM (2005). Reporting recommendations for tumor marker prognostic studies (REMARK). J. Natl. Cancer Inst..

[49] Caporale A (2010). Quantitative investigation of desmoplasia as a prognostic indicator in colorectal cancer. J. Investig. Surg..

[50] Caporale A (2005). Is desmoplasia a protective factor for survival in patients with colorectal carcinoma?. Clin. Gastroenterol. Hepatol..

[51] Zippi M (2017). Desmoplasia influenced recurrence of disease and mortality in stage III colorectal cancer within five years after surgery and adjuvant therapy. Saudi J. Gastroenterol..

[52] Coulson-Thomas VJ (2011). Colorectal cancer desmoplastic reaction up-regulates collagen synthesis and restricts cancer cell invasion. Cell Tissue Res..

[53] Jensen C (2018). Serum type XVI collagen is associated with colorectal cancer and ulcerative colitis indicating a pathological role in gastrointestinal disorders. Cancer Med..

[54] Rolff HC (2016). The prognostic and predictive value of soluble type IV collagen in colorectal cancer: a retrospective multicenter study. Clin. Cancer Res..

[55] van Huizen NA (2019). Up-regulation of collagen proteins in colorectal liver metastasis compared with normal liver tissue. J. Biol. Chem..

[56] van Haaften WT (2017). Misbalance in type III collagen formation/degradation as a novel serological biomarker for penetrating (Montreal B3) Crohn’s disease. Aliment. Pharmacol. Ther..

[57] Lipton A (2018). High turnover of extracellular matrix reflected by specific protein fragments measured in serum is associated with poor outcomes in two metastatic breast cancer cohorts. Int. J. Cancer.

[58] Tong G (2018). The role of tissue and serum carcinoembryonic antigen in stages I to III of colorectal cancer-A retrospective cohort study. Cancer Med..

[59] Thomsen M (2018). Prognostic role of carcinoembryonic antigen and carbohydrate antigen 19–9 in metastatic colorectal cancer: A BRAF-mutant subset with high CA 19–9 level and poor outcome. Br. J. Cancer.

[60] Wang J (2015). Combined detection of preoperative serum CEA, CA19-9 and CA242 improve prognostic prediction of surgically treated colorectal cancer patients. Int. J. Clin. Exp. Pathol..

[61] Rosen LS, Jacobs IA, Burkes RL (2017). Bevacizumab in colorectal cancer: current role in treatment and the potential of biosimilars. Target. Oncol..

[62] Schober M (2014). Desmoplasia and chemoresistance in pancreatic cancer. Cancers (Basel)..

[63] Mariathasan S (2018). TGFβ attenuates tumour response to PD-L1 blockade by contributing to exclusion of T cells. Nature.

[64] Ciardiello D, Elez E, Tabernero J, Seoane J (2020). Clinical development of therapies targeting TGFβ: current knowledge and future perspectives. Ann. Oncol..

[65] Mooi JK (2018). The prognostic impact of consensus molecular subtypes (CMS) and its predictive effects for bevacizumab benefit in metastatic colorectal cancer: Molecular analysis of the AGITG MAX clinical trial. Ann. Oncol..

[66] Menter DG (2019). Back to the colorectal cancer consensus molecular subtype future. Curr. Gastroenterol. Rep..

[67] Tang Y-Q (2020). The tumor immune microenvironment transcriptomic subtypes of colorectal cancer for prognosis and development of precise immunotherapy. Gastroenterol. Rep..

[68] Guinney J (2015). The consensus molecular subtypes of colorectal cancer. Nat. Med..

[69] Nielsen MJ (2013). The neo-epitope specific PRO-C3 ELISA measures true formation of type III collagen associated with liver and muscle parameters. Am. J. Transl. Res..

