# Supplementary material for: Prognostic value of blood-based fibrosis biomarkers in patients with metastatic colorectal cancer receiving chemotherapy and bevacizumab
Source: Sci Rep. 2021 Jan 13;11:865. doi: 10.1038/s41598-020-79608-0 (PMC7806753; doi:10.1038/s41598-020-79608-0)
Supplement: Supplementary file 1 — Supplementary Information. [file 41598_2020_79608_MOESM1_ESM.pdf]

Supplementary figures

Prognostic value of blood-based fibrosis biomarkers in patients with metastatic colorectal cancer receiving chemotherapy and bevacizumab

Neel I. Nissen[1,2#*], Stephanie Kehlet[2#], Mogens K. Boisen[3], Maria Liljefors[4], Christina Jensen[2], Astrid Z. Johansen[3], Julia S. Johansen[3,5,6], Janine T. Erler[1], Morten Karsdal[2], Joachim H. Mortensen [2], Anette Høye[1], and Nicholas Willumsen[2]

[#]authors contributed equally to the work

[*]*Corresponding author:* Neel Ingemann Nissen, Herlev Hovedgade 205-207 2730 Herlev, Denmark. +45 4452 5252, nin@nordicbio.com

[1]Biotech Research & Innovation Centre (BRIC), University of Copenhagen (UCPH), Denmark.

[2] Biomarkers & Research, Nordic Bioscience, Herlev, Denmark.

[3] Department of Oncology, Herlev and Gentofte Hospital, Copenhagen University Hospital, Denmark.

[4] Department of Clinical Science, Intervention and Technology, Karolinska University Hospital Huddinge, Stockholm, Sweden

[5] Department of Medicine, Herlev and Gentofte Hospital, Copenhagen University Hospital, Denmark.

[6] Department of Clinical Medicine, Faculty of Health and Medical Sciences, University of Copenhagen, Denmark

Figure S1: Assessment of the prognostic potential of low and high (median) biomarker levels in patients in first line chemotherapy a) PRO-C3 (formation of collagen type III), b) PRO-C6 (formation of collagen type VI), c) C6M (degradation of collagen type VIα1) and d) C6Mα3 (degradation of collagen type VIα3) serum levels by Kaplan Meier plots. Cox proportional-hazards regression was used to calculate the hazard ratios (HR) with 95% CI and p-values. A $p<0.05$ was considered significant.

Figure S2: Assessment of the prognostic potential of low and high (median) biomarker levels in patients in second or later line of chemotherapy a) PRO-C3 (formation of collagen type III), b) PRO-C6 (formation of collagen type VI), c) C6M (degradation of collagen type VIα1) and d) C6Mα3 (degradation of collagen type VIα3) serum levels by Kaplan Meier plots. Cox proportional-hazards regression was used to calculate the hazard ratios (HR) with 95% CI and p-values. A $p<0.05$ was considered significant.

Fig S1:

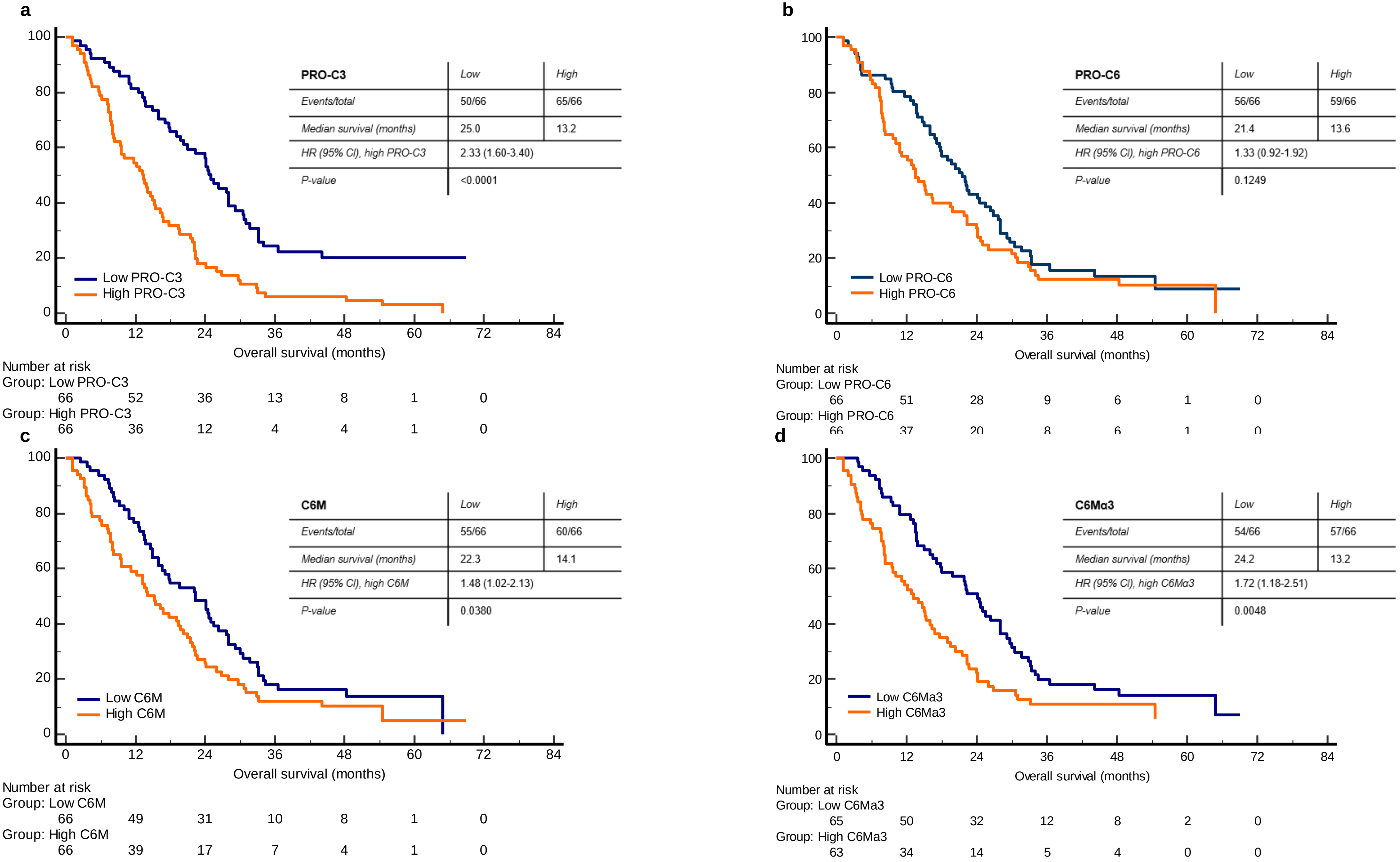
a
PRO-C3 | Low | High
Events/total | 50/66 | 65/66
Median survival (months) | 25.0 | 13.2
HR (95% CI), high PRO-C3 | 2.33 (1.60-3.40)
P-value | <0.0001
Low PRO-C3
High PRO-C3
Overall survival (months)
Number at risk
Group: Low PRO-C3
66 52 36 13 8 1 0
Group: High PRO-C3
66 36 12 4 4 1 0
b
PRO-C6 | Low | High
Events/total | 56/66 | 59/66
Median survival (months) | 21.4 | 13.6
HR (95% CI), high PRO-C6 | 1.33 (0.92-1.92)
P-value | 0.1249
Low PRO-C6
High PRO-C6
Overall survival (months)
Number at risk
Group: Low PRO-C6
66 51 28 9 6 1 0
Group: High PRO-C6
c
C6M | Low | High
Events/total | 55/66 | 60/66
Median survival (months) | 22.3 | 14.1
HR (95% CI), high C6M | 1.48 (1.02-2.13)
P-value | 0.0380
Low C6M
High C6M
Overall survival (months)
Number at risk
Group: Low C6M
66 49 31 10 8 1 0
Group: High C6M
66 39 17 7 4 1 0
d
C6Mα3 | Low | High
Events/total | 54/66 | 57/66
Median survival (months) | 24.2 | 13.2
HR (95% CI), high C6Mα3 | 1.72 (1.18-2.51)
P-value | 0.0048
Low C6Ma3
High C6Ma3
Overall survival (months)
Number at risk
Group: Low C6Ma3
65 50 32 12 8 2 0
Group: High C6Ma3
63 34 14 5 4 0 0

Fig S2:

a

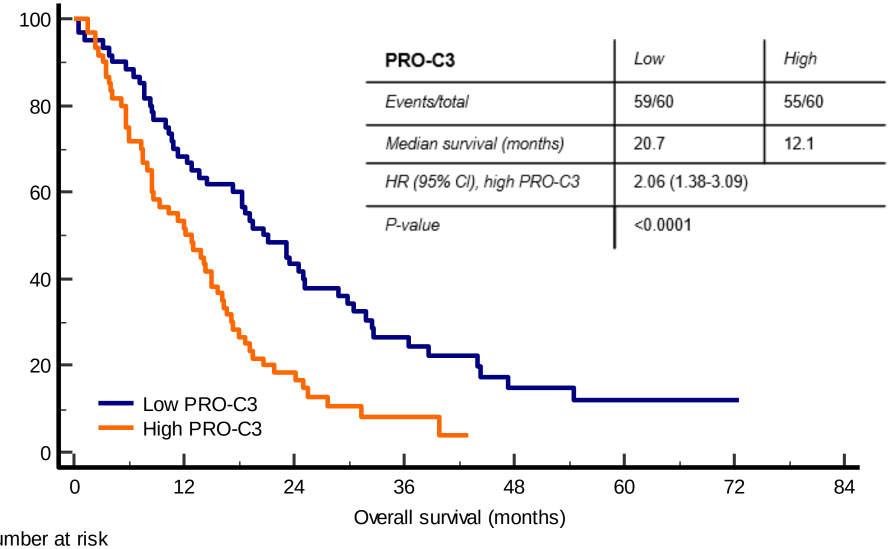

Number at risk

Group	0	12	24	36	48	60	72	84
Group: Low PRO-C3	60	41	26	12	5	4	1	0
Group: High PRO-C3	60	31	10	3	0	0	0	0

b

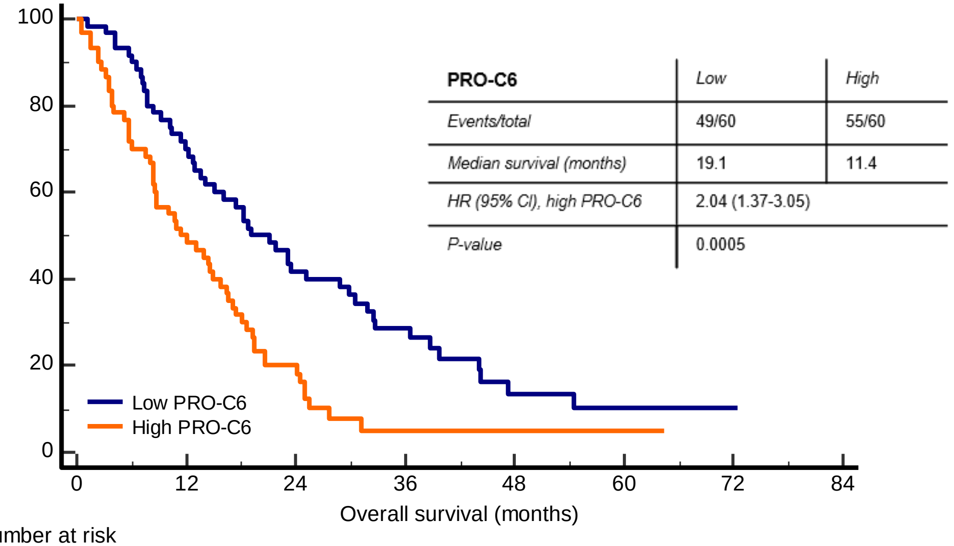

Number at risk

Group	0	12	24	36	48	60	72	84
Group: Low PRO-C6	60	42	25	13	4	3	1	0
Group: High PRO-C6	60	30	11	2	1	1	0	0

c

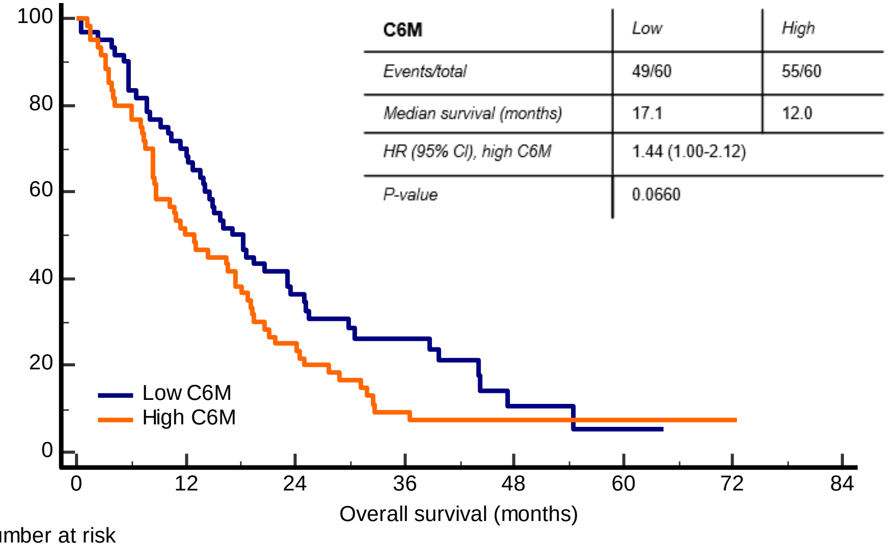

Number at risk

Group	0	12	24	36	48	60	72	84
Group: Low C6M	60	42	21	10	2	1	0	0
Group: High C6M	60	30	15	5	3	3	1	0

d

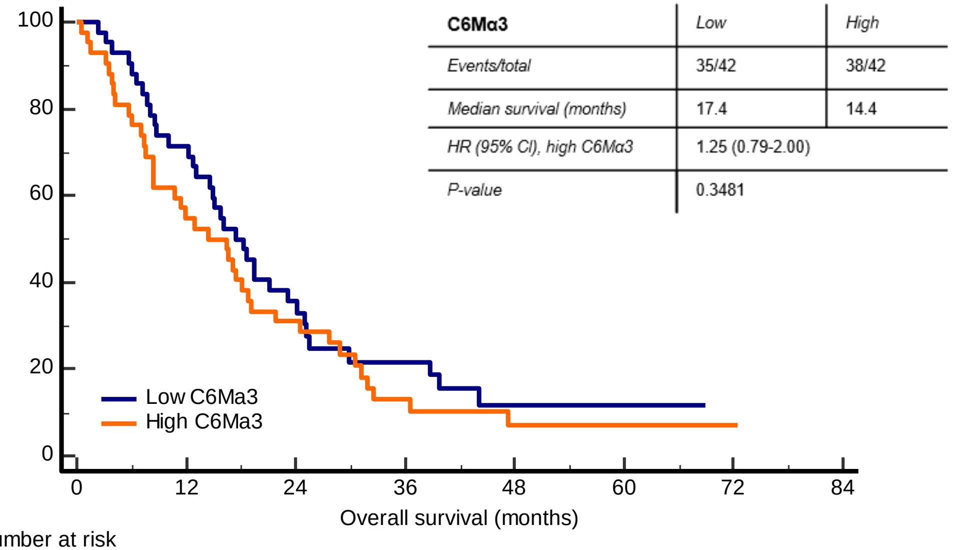

Number at risk

Group	0	12	24	36	48	60	72	84
Group: Low C6Ma3	42	30	14	7	2	2	0	0
Group: High C6Ma3	42	23	13	5	2	2	1	0
